# Tomato Divinyl Ether-Biosynthesis Pathway Is Implicated in Modulating of Root-Knot Nematode *Meloidogyne javanica*'s Parasitic Ability

**DOI:** 10.3389/fpls.2021.670772

**Published:** 2021-08-25

**Authors:** Payal Sanadhya, Anil Kumar, Patricia Bucki, Nathalia Fitoussi, Mira Carmeli-Weissberg, Menachem Borenstein, Sigal Brown-Miyara

**Affiliations:** ^1^Department of Entomology, Nematology and Chemistry Units, Agricultural Research Organization, The Volcani Center, Bet Dagan, Israel; ^2^Department of Plant Pathology and Microbiology, The Faculty of Agriculture, Food and Environment, The Hebrew University of Jerusalem, Rehovot, Israel; ^3^Metabolomics, Institute of Plant Sciences, Agricultural Research Organization, The Volcani Center, Bet Dagan, Israel; ^4^Department of Plant Pathology and Weed Research, Agricultural Research Organization (ARO), The Volcani Center, Bet Dagan, Israel

**Keywords:** divinyl ether synthase, *Meloidogyne javanica*, plant defense signaling, oxylipins, hormone signaling, innate immunity

## Abstract

The role of the 9-lipoxygenase (9-LOX)-derived oxylipins in plant defense is mainly known in solanaceous plants. In this work, we identify the functional role of the tomato divinyl ether synthase (LeDES) branch, which exclusively converts 9-hydroperoxides to the 9-divinyl ethers (DVEs) colneleic acid (CA) and colnelenic acid (CnA), during infection by the root-knot nematode *Meloidogyne javanica*. Analysis of *LeDES* expression in roots indicated a concurrent response to nematode infection, demonstrating a sharp increase in expression during the molting of third/fourth-stage juveniles, 15 days after inoculation. Spatiotemporal expression analysis using an *LeDES promoter:GUS* tomato line showed high GUS activity associated with the developing gall; however the GUS signal became more constricted as infection progressed to the mature nematode feeding sites, and eventually disappeared. Wounding did not activate the LeDES promoter, but auxins and methyl salicylate triggered *LeDES* expression, indicating a hormone-mediated function of DVEs. Heterologous expression of *LeDES* in *Arabidopsis thaliana* rendered the plants more resistant to nematode infection and resulted in a significant reduction in third/fourth-stage juveniles and adult females as compared to a vector control and the wild type. To further evaluate the nematotoxic activity of the DVEs CA and CnA, recombinant yeast that catalyzes the formation of CA and CnA from 9-hydroperoxides was generated. Transgenic yeast accumulating CnA was tested for its impact on *M. javanica* juveniles, indicating a decrease in second-stage juvenile motility. Taken together, our results suggest an important role for *LeDES* as a determinant in the defense response during *M. javanica* parasitism, and indicate two functional modes: directly via DVE motility inhibition effect and through signal molecule-mediated defense reactions to nematodes that depend on methyl salicylate.

## Introduction

Root knot nematodes (RKNs) are devastating pathogens of a wide variety of plants, causing substantial annual losses in yield worldwide (McCarter, 2009). They also incur the intensive use of toxic nematicides that pose a great threat to human and environmental health (Hague and Gowen, 1987; Johnson and Feldmesser, 1987; Thomason, 1987; Desaeger et al., 2020). The consequent withdrawal of frontline nematicides has put a significant dent in our ability to control RKNs, as well as all other plant-parasitic nematodes. The *Meloidogyne* life cycle consists of six stages: eggs, J1 (first-stage juvenile), J2 (second-stage juvenile), J3 (third-stage juvenile), J4 (fourth-stage juvenile), and adults (female and male). The J2, which are motile and infective, pierce the elongation zone of the plant root near its tip and migrate intercellularly through the root apex to the vascular cylinder (Bird et al., 2009). Once they reach the differentiation zone in the vascular bundle, they become sessile and induce the transformation of 4–8 root cells into coenocytic giant cells that operate as feeding sites, supplying the RKNs with the nutrients required for their development and reproduction (Wyss et al., 1992; Davis et al., 2004; Bird et al., 2009).

Transcriptomic studies of *Arabidopsis thaliana, Glycine max* (soybean), and *Solanum lycopersicum* (tomato) plants in response to RKN infection have revealed complicated multistep signaling cascades that are instigated by pathogen attack (Bar-Or et al., 2005; Jammes et al., 2005; Alkharouf et al., 2006; Szakasits et al., 2009). Among them, fatty acid signaling produces oxygenated fatty acids known as oxylipins, which are involved in defense against pathogen attack (Feussner and Wasternack, 2002). Studies have shown that various oxylipin-biosynthesis pathway genes are induced in plants by pathogen attack (Deboever et al., 2020; Estelle et al., 2020), along with a spike in the concentration of related oxylipins as shown in oxylipin-profiling studies (Weber et al., 1999; Göbel et al., 2001; Mariutto et al., 2014; Fitoussi et al., 2021). Several studies on mutant or transgenic plants with altered synthesis or signaling of oxylipins have suggested a direct signaling role for oxylipins in the plant's response to pathogen attack (Deboever et al., 2020), acting as antimicrobial (Croft et al., 1993; Weber et al., 1999; Granér et al., 2003; Prost et al., 2005) or nematicidal agents (Naor et al., 2018). Overexpression of jasmonic acid carboxyl methyltransferase in *Arabidopsis* or 9-lipoxygenase (9-LOX) in tobacco conferred resistance against the necrotrophic pathogen *Botrytis cinerea* (Seo et al., 2001) and the oomycete pathogen *Phytophthora parasitica* (Mène-Saffrané et al., 2003). Ozalvo et al. (2014) also demonstrated the role of 13-LOXs in *Arabidopsis*; they found that LOX3 and LOX4 are involved in the response to nematodes, and that LOX4 is required for resistance to nematode infection. High expression levels of the divinyl ether synthase (*DES*) and *9-LOX* genes was also detected in pepper (*Capsicum annuum*) leaves inoculated with *Obuda pepper virus* (Gullner et al., 2010; Juhász et al., 2015). Moreover, disruption of *LOX1* in pepper plants renders them more susceptible to *Xanthomonas campestris* pv. *vesicatoria* and *Colletotrichum coccodes* infection, further emphasizing the role of LOX-biosynthesis pathway products in pepper plants defense response (Hwang and Hwang, 2010).

Infection of potato leaves by the pathogens *Phytophthora infestans* and *Pseudomonas syringae* leads to elevated transcription of *DES* and accumulation of the 9-divinyl ethers (DVEs) colneleic acid and colnelenic acid (CA and CnA, respectively; Weber et al., 1999; Stumpe et al., 2001). The involvement of CA and CnA in plant defense was also demonstrated by Fammartino et al. (2007), who reported high concentrations of CA and CnA in wild-type (WT) tobacco roots challenged with *P. parasitica* var. *nicotianae*, whereas silencing of *NtLOX1* downregulated the production of CA and CnA. The *DES* genes involved in the synthesis of CA and CnA in potato and tomato have been cloned and characterized (Itoh and Howe, 2001; Stumpe et al., 2001). 9-Divinyl ethers have an important role in defense mechanisms against different plant pathogens and can act as antimicrobial agents (Weber et al., 1999; Göbel et al., 2001; Stumpe et al., 2001; Granér et al., 2003; Fammartino et al., 2007) or signaling components that induce further defense responses. Although much literature is available on the structural diversity of DVEs (Göbel and Feussner, 2009) their occurrence in different plants and their role as antimicrobial agents (Weber et al., 1999; Prost et al., 2005; Fammartino et al., 2007); information on transcriptional regulation of *DES* and its potential role in mediating defense responses is rather scarce, with no knowledge of its function in the tomato–RKN system. Here, we investigated the functional role of *DES* from tomato (*S. lycopersicum*) in response to nematode infection. To further study the spatial and temporal expression of *LeDES*, we generated *pLeDES::GUS* transgenic hairy root lines which show induced expression of *LeDES* in response to early and middle–late stages (15 days) of *Meloidogyne javanica* infection. In addition, the full-length *LeDES* was functionally characterized by heterologous expression in *Arabidopsis* plants. Characterization of transgenic lines indicated a significant reduction in the number of *M. javanica* developmental stages and suggests a role for the DVEs in modulating *M. javanica* disease development in the host root.

## Materials and Methods

### Plant Materials and Growth Conditions

Tomato (*Lycopersicon esculentum*) cv Avigail 870 was used as the background line for the transformation in experiments involved tomato plants. For the transformation protocol, tomato seeds were treated with 1.6% NaOCl for 10 min with continuous shaking, washed with sterile water for 5 min, and placed on standard-strength Gambourg's B5 medium supplemented with 2% sucrose and 0.8% Gelrite agar (Duchefa, Haarlem, The Netherlands). The plates were kept in darkness at 26°C for 2 days, followed by 2 weeks under a 16/8-h photoperiod until cotyledons emerged. They then were used immediately for cocultivation as described by Chinnapandi et al. (2017). Similarly, tomato roots sections were subculture, by placing one root per Petri dish (Miniplast, Ein Shemer, Israel), containing B5 medium. Branching roots were used for inoculation as described below. For experiments conducted on Arabidopsis, *A. thaliana* WT and transgenic lines were grown in a growth chamber at 24°C on MS medium (Murashige and Skoog, 1962) or in pots filled with cocopeat. The plants were maintained under a 14 h/10 h day/night cycle at a light intensity of 50–60 μmol/m^2^s. *Arabidopsis thaliana* ecotype Columbia-0 (Col-0) was used as the genetic background for all transgenic lines.

### Nematode Inoculation and Infection of Tomato and Arabidopsis Plants

*Meloidogyne javanica* was propagated on greenhouse-grown tomato “Avigail” (870) plants. Nematode egg masses were extracted from roots with 0.05% (v/v) sodium hypochlorite followed by sucrose flotation (Hussey and Baker, 1973). For sterilization, eggs were placed on a cellulose–acetate filter membrane (Sartorius Stedim Biotech GmbH, Goettingen, Germany, pore size 5 μm) in a sterile Whatman® filter holder (Whatman International Ltd., Dassel, Germany). Eggs on the filter were exposed for 10 min to 0.01% (w/v) mercuric chloride (Sigma-Aldrich, St Louis, MO, USA), followed by 0.7% (v/v) streptomycin solution (Sigma-Aldrich), and three washing steps with 50 ml sterilized distilled water (Jansen van Vuuren and Woodward, 2001). The sterilized eggs were collected from the membrane and placed on 25-μm-pore sieves in 0.01 M 2-morpholinoethanesulfonic acid buffer (Sigma-Aldrich) under aseptic dark conditions for 3 days, allowing J2s to hatch. Freshly hatched preparasitic J2s were collected in a 50 ml falcon tube. For nematode infection, 1-week-old transgenic tomato root hairy root lines, growing on standard-strength Gamborg's B5 salt medium, were inoculated with 200 sterile freshly hatched *M. javanica* preparasitic J2s. Plates were left uncovered in a laminar flow hood until water had completely soaked into the medium (Sijmons et al., 1991). The inoculated and non-inoculated roots were incubated horizontally in the dark, and root samples were taken for GUS bioassay at the designated time points after wounding or inoculation. To test the response of transgenic Arabidopsis plants to nematode infection, three transgenic lines were used, 10 plants per line in each experiment. Seeds of WT, vector-only and transgenic *Arabidopsis* lines were surface sterilized and germinated on standard-strength GB media with 2% (w/v) sucrose and 0.8% (w/v) Gelrite as described previously by Joshi et al. (2019). One-week-old seedlings grown *in vitro* were gently transferred with a forceps to a petri dish containing GB media, one plant per dish. Each seedling was inoculated with 300 sterile freshly hatched *M. javanica* J2s applied near the roots; the plates were left open until the solution was completely absorbed into the media (Sijmons et al., 1991). The infected seedlings were kept vertically and root samples were gently harvested 28 days post-infection (dpi).

### *LeDES* Transcript Accumulation, RNA Isolation, and Quantitative RT-PCR (qRT-PCR) Analysis

Expression of *LeDES* in infected tomato roots (non-transgenic) at different time points was analyzed by qRT-PCR using gene-specific primers LeDes-rtF: 5′-CCGGATGAGTTTGTACCTGA-3′ and LeDes-rtR: 5′-ATCTTTGCCTGGACATTGCT-3′(López-Ráez et al., 2010). Ten root systems (100 mg) from each time point were pooled from uninfected and infected roots. Total RNA was extracted using Trizol reagent (Invitrogen, Carlsbad, CA, USA) and subjected to Turbo DNase (Ambion, Thermo Fisher Scientific, Waltham, MA, USA). One microgram of total RNA was reverse-transcribed to cDNA using the Verso cDNA Synthesis Kit (Thermo Scientific, Waltham, MA, USA) according to the manufacturer's instructions; qRT-PCR was carried out in a StepOne Real Time PCR system (Applied Biosystems, Foster City, CA, USA) in 10 μl reaction mix comprised of 3.4 μl cDNA, SYBR-Green ROX Mix (Abgene, Portsmouth, NH, USA), 150 nM forward primer and 150 nM reverse primer as described in Chinnapandi et al. (2019). Reactions were performed in MicroAmp 96-well plates (Applied Biosystems) and PCR cycles consisted of initial denaturation at 95°C for 10 min; 40 cycles at 95°C for 15 s, 56°C for 20 s, and 72°C for 20 s, and a final extension at 72°C for 2 min. Reactions were conducted in triplicate, with a control with no template, and the presented results are the means of two independent biological experiments. The qRT-PCR results were analyzed and interpreted using the 2^−ΔΔCt^ method (Livak and Schmittgen, 2001) integrated into StepOne v2.3 of the StepOne Plus (Applied Biosystems) real-time PCR instrument. Tomato β-*tubulin* gene (GenBank accession number NM_001247878.1) was used as the internal reference to normalize the expression of *LeDES*.

### Plasmid Construction and Generation of pLeDES::GUS Reporter Hairy-Root Cultures

Genomic DNA was isolated from soil-grown tomato seedlings, cv. Avigail 870, according to the cetyltrimethylammonium bromide (CTAB) method (Murray and Thompson, 1980). A promoter–GUS construct, to study the expression pattern of *LeDES*, was generated by Gateway Technology (Invitrogen). A 1,602-bp sequence upstream of the ATG start codon of *LeDES* was amplified with Platinum Taq DNA polymerase (Invitrogen) using the primers LeDESprom (attB1) 5′-GGGGACAAGTTTGTACAAAAAAGCAGGCT-GTATAGTGTAGCTACGCGCTT-3′ and LeDESprom (attB2) 5′-GGGGACCACTTTGTACAAGAAAGCTGGGT-TTTTCTTAACAAGTTTTGG-3′ and tomato genomic DNA as the template. The purified product was first cloned into the *pDONR221* vector (Invitrogen) by BP reaction, generating the entry vector. The entry vector was then cloned by LR Clonase II (Thermo Fisher Scientific) into the destination vector pKGWFS7 (VIB-Ugent Center for Plant System Biology, Ghent, Belgium) upstream of *GFP::GUS*-coding sequences, generating the *pKGWFS7:prom-LeDES* (*pLeDES::GUS*) construct. The resulting promoter construct was confirmed by sequencing prior to *Rhizobium rhizogenes* transformation. The *pLeDES::GUS* construct was mobilized to *Rhizobium rhizogenes* ATCC 15834 by freeze–thaw procedure as described by Xu and Li (2008). The transformed *R. rhizogenes* colonies were confirmed by colony PCR using LeDES promoter-specific primers (pLeDESF: 5′-GTATAGTGGAGCTCCGCGCTT-3′ and pLeDESR: 5′-CCCCCGGGTTTTCTTAACAAGTTTTGG-3′) and kanamycin primers (kanF: 5′-GCTCTTCGTCCAGATCATCC-3′ and kanR: 5′-GCGTTCAAAAGTCGCCTAAG-3′).

*R. rhizogenes*-mediated transformation of tomato cotyledons was carried out according to the protocol described by Chinnapandi et al. (2017). Two weeks after transformation, cotyledons with emerging kanamycin-resistant roots were transferred to Gamborg's B5 salts medium (GB; Duchefa Biochemie, Haarlem, The Netherlands) containing 0.8% (w/v) Gelrite + cefotaxime (200 mg/L) + kanamycin (50 mg/L). Roots were excised from the cotyledons once they were 1.0 cm long. The excised roots were transferred to individual GB medium plates (with 200 mg/L cefotaxime + 50 mg/L kanamycin). After two rounds of subculture, cefotaxime was eliminated from the media and transgenic roots were maintained on GB medium plates (with 50 mg/L kanamycin) for further analysis. The genomic DNA of the hairy roots was isolated and used as a template for identification of the positive transgenic lines with promoter-specific (*pLeDESF* and *pLeDESR*) and kanamycin (*kanF* and *kanR*) primers.

### Histochemical Localization of GUS Activity and Microscopic Analysis

For the wounding treatment, transgenic roots were subcultured in GB media for 1 week. Roots were mechanically wounded at several points across their length using sterile forceps. Wounded roots were kept on the GB plates in the dark and collected after 6 and 24 h. GUS activity was assessed histochemically by incubating root samples in GUS staining buffer: 50 mM sodium phosphate (pH 7.0), 10 mM EDTA, 5 mM K_4_[Fe_2_(CN)_6_], 5 mM K_3_[Fe_2_(CN)_6_], 0.2% (v/v) Triton X-100, and 2 mM 5-bromo-4-chloro-3-indolyl ß- D-glucuronide (X-Gluc) overnight at 37°C as described previously (Iberkleid et al., 2015). The stained roots were washed twice with distilled water, and then suspended in water in petri plates or mounted on slides and observed under a Leica DMLB light microscope (Leica Microsystems GmbH, Wetzlar, Germany) and photographed with a Nikon Eclipse 90i (Nikon Corporation, Tokyo, Japan), or observed under a stereomicroscope (Leica MZFLIII, Leica Microsystems GmbH) equipped with a Nikon DS-Fi1 camera.

To study the effect of hormones on the expression of *LeDES*, 1-week-old pLeDES::GUS roots were subcultured on a GB plate supplemented with indoleacetic acid (IAA; 1 and 5 μM), indolebutyric acid (IBA; 1 and 10 μM), methyl jasmonate (Me-JA; 0.01 and 0.1 mM), or methyl salicylate (Me-SA; 1 and 5 mM) for 16 h. One-week-old roots grown on GB medium were used as a control. Treated roots were harvested and analyzed by GUS staining as described above.

Prior to the infection assay with *M. javanica* J2s, the established root cultures were transferred to antibiotic-free GB media for 1 week. Control and *pLeDES::GUS* root cultures were inoculated with 300 J2s per culture previously sterilized as described above. Non-infected transgenic roots served as the control. The inoculated and non-inoculated transgenic roots were placed in the dark, and the roots were gently harvested for analysis of histochemical GUS activity at designated time points post-inoculation.

For tissue localization of GUS signal within galls, stained galls were dissected, fixed in 1% (w/v) glutaraldehyde and 4% (v/v) formaldehyde in 50 mM sodium phosphate buffer pH 7.2, dehydrated, and embedded in Technovit 7100 (Heraeus Kulzer, Wehrheim, Germany) according to the manufacturer's instructions. Semi-thin sections (3 μm) were cut, mounted in DePeX (Sigma-Aldrich) and observed under a Nikon DS Ri2-equipped microscope with dark-field illumination (Leica Microsystems GmbH).

### Plasmid Construction and *Agrobacterium tumefaciens*-Mediated Transformation of *Arabidopsis* Plants

For amplification of *LeDES*, PCR was performed with cDNA from 1-month-old tomato plants as the template. The full-length *LeDES* (1,437 bp) coding sequence was amplified using gene-specific primers (*LeDESF* 5′-GTATGGGTACCATGGATACAAA CTTGG-3′ and *LeDESR* 5′-CCTAGAAGCTTTTATATAATTTTTTGCATTTGA-3′), with *Kpn*I and *Hind*III restriction enzyme cutting sites in the forward and reverse primers, respectively. The PCR products were digested with *Kpn*I/*Hind*III and ligated into the *pHANNIBAL* vector. The expression cassette (3,700 bp) containing *LeDES* flanked by the *CaMV35S* promoter and *OCS* terminator was obtained as fallout after digestion with *Not*I restriction enzyme, which was subsequently cloned into the *pART27* binary vector to generate expression plasmid *pART27::LeDES*. The identity, orientation, and vector-insert junctions for *pART27::LeDES* were checked by restriction enzyme digestion and sequencing. The *pART27::LeDES* and *pART27* empty vector were transferred into *Agrobacterium tumefaciens* strain GV3101 by freeze–thaw method as described in Xu and Li (2008). Positive *Agrobacterium* transformants containing *pART27::LeDES* and *pART27* were transformed into *Arabidopsis* Col-0 plants (5-week-old flowering plants) by floral dipping method (Clough and Bent, 1998). The T_1_ transformants were screened on agar plates containing half-strength MS medium supplemented with 50 mg/L kanamycin. Segregation ratios of T_2_ lines with kanamycin resistance were calculated and lines showing an insertional event were selected. Independent T_3_ plants homozygous for kanamycin resistance were used for the infection experiments. The presence of *LeDES* was confirmed by PCR on genomic DNA isolated from the transgenic lines. Expression of *LeDES* for each homozygous line was determined by RT-PCR using cDNA.

### DVE Extraction and Analysis by LC–MS

Analysis for the presence of CnA and CA in transgenic *Arabidopsis* roots was performed according to method described by Kuroda with minor modifications (Kuroda et al., 2005). Production of CnA and CA from (9S)-hydroperoxy linoleic acid (9-HPODE; Cayman Chemicals, Ann Arbor, MI, USA) by root lysate of transgenic *Arabidopsis* lines (D1, D2, and D3) and control lines was measured by LC–MS as follows. *Arabidopsi*s root samples were harvested 2 weeks after germination and homogenized in Bead Ruptor Elite (OMNI International, Kennesaw, GA, USA) and 500 μl of 50 mM sodium phosphate buffer pH 7. Samples were centrifuged at 15,000 g and the supernatant was collected and incubated with 5 μg/ml 9-HPODE at 30°C overnight. The reaction was terminated by adding an equal amount of 1 ppm 2-hydroxydecanoic acid (Cayman Chemicals) in ethanol. LC–MS analyses were conducted using a UPLC-Triple Quadrupole mass spectrometer (Waters Xevo TQ MS, Waters Corp., Milford, MA, USA). Separation was performed on a Waters Acquity UPLC BEH C18 1.7 μm, 2.1 × 100 mm column with a VanGuard precolumn (BEH C18 1.7 μm, 2.1 × 5 mm). Chromatographic and MS parameters were as follows: the mobile phase consisted of water (phase A) and acetonitrile (phase B), both containing 0.1% (v/v) formic acid in gradient elution mode. The flow rate was 0.3 ml/min, and the column temperature was kept at 35 °C. LC–MS analysis was performed using the ESI source in positive ion mode for CA and CnA and in negative ion mode for 2-hydroxydecanoic acid with the following settings: capillary voltage 3.1 kV, cone voltage 30 V, desolvation temperature 350°C, desolvation gas flow 650 L/h, source temperature 150°C. Quantification was performed using MRM acquisition by monitoring the 293/275, 293/93 [retention time (RT) = 9.50, dwell time of 100 ms for each transition] for CnA, 295/81, 295/277 (RT = 10.08, dwell time of 100 ms) for CA, 187/141, 187/169 (RT = 6.51, dwell time of 161 ms) for 2-hydroxydecanoic acid. Acquisition of LC–MS data was performed using MassLynx v4.1 software (Waters).

### Evaluation of *Arabidopsis* Plant Response to RKN Infection

To study the extent of nematode infection in WT, vector-only, and transgenic *Arabidopsis* lines, infected roots grown in monoxenic culture were collected at 28 dpi and analyzed following the procedure describe by Joshi et al. (2019, 2020) with minor modifications. Roots were gently harvested from the plates, agar was removed, and they were weighed. After cleaning, the roots were stained with acid fuchsin (Sigma-Aldrich) solution (17.5 mg acid fuchsin, 500 ml ethanol, 500 ml acetic acid) overnight. Stained roots were washed three times with distilled water, then mounted in water, and roots and galls were manually dissected under a stereomicroscope (Olympus SZX12, Tokyo, Japan). Different nematode development stages (J3/J4 and adult females) embedded in the roots and galls were manually dissected and counted for each root. Each treatment (Arabidopsis line) included 10 individual plates and the infection assays were repeated three times. Infection assay data were fitted with general linear mixed model in SPSS, by keeping samples as fixed factors and repeated assays as random factor. Further, Tukey *post-hoc* range test was applied to check significant differences (*p* < 0.05) among different samples.

### Heterologous Expression of *LeDES* in Yeast

Full-length *LeDES* cDNA was cloned into the yeast expression vector pFL61 under the control of the constitutive yeast phosphoglycerate kinase (PGK) promoter (Minet et al., 1992). To generate yeast expression of the construct, *LeDES* cDNA was amplified from *pART27::LeDES* using *Not*I-containing primers (*DesNotF*: 5′-TTGCGGCCGCATGTCTTCTTATTCAGAG-3′ and *DesNotR*: 5′-AAGCGGCCGCCTATTTACTTGCTTTAGT-3′) and cloned into the pFL61 vector. *Saccharomyces cerevisiae* INVSc1 (MATa his3Δ1 leu2 trp1-289 ura3-52/MATα his3Δ1 leu2 trp1-289 ura3-52), procured from Invitrogen, was used as the host strain for yeast transformation. *S. cerevisiae* was transformed with the empty pFL61 vector (negative control) and with the *pFL61::LeDES* construct using the lithium acetate/polyethylene glycol transformation method (Sanadhya et al., 2015). Positive colonies were selected on synthetic dropout (SD) plates with synthetic defined medium lacking uracil (SD/-Ura; Clontech, Mountain View, CA, USA), and verified by PCR using gene-specific primers. Expression of *LeDES* in transgenic yeast clones was further confirmed by RT-PCR using cDNA.

### Evaluating the Immotility Effect of Transgenic Yeast on J2s

The method used in this study to analyze the impact of the transgenic yeast harboring *LeDES* was adapted from Naor et al. (2018) with some minor modifications. *S. cerevisiae* cells transformed with *pFL61* and *pFL61::LeDES* were grown overnight at 28°C from individual colonies in 5 ml of SD/-Ura medium. The precultured yeast cells were pelleted by centrifugation and resuspended in 10 ml of induction medium: SD/-Ura with or without 9-HPODE or 13-HPODE to a final optical density at 600 nm of 2.0. After overnight incubation at 28°C with the respective oxylipin, 300 sterile J2s suspended in 0.01 M MES buffer were added to the cultures and incubated for additional 24 h at 25°C with shaking at 30 rpm. After 24 h incubation, the J2s in the suspensions were collected and subjected to 30-μm filtering (AD Sinun Technologies, Petach Tikvah, Israel) for 2 h (Sanadhya et al., 2018). J2s that actively passed through the filter were counted with nematode-counting slides (Chalex LLC, Portland, OR, USA) under the microscope (Wilovert Standard microscope, Helmut Hund GmbH, Wetzlar, Germany). To measure % motility, J2s counting in treatments included *pFL61* or *pFL61::LeDES* plasmids and the oxylipins 9-HPODE or 13-HPODE were divided by J2s counts following incubation with respective plasmids but without the respective oxylipins. Thus, the pure nematotoxic activity of *LeDES* could be assessed. Ten replicates were used for each treatment and experiments were performed three times independently. A general linear mixed model ANOVA test was applied to the data with similar results. Different letters above the columns indicate significant difference (*P* ≤ 0.05) among the different treatments analyzed by Tukey *post-hoc* range test.

### Sample Preparation From Transgenic Yeast for DVE Extraction and Analysis by LC–MS

colnelenic acid (CnA) and colneleic acid (CA) were produced from 9-HPODE by yeast lysate of transgenic and control yeast lines according to the method described by Kuroda with minor modifications (Kuroda et al., 2005). Yeast strains carrying empty vector and *LeDES* were precultured in SD/-Ura liquid medium at 28°C. After 16 h, the starter culture was added to a 250-ml flask containing 50 ml SD/-Ura liquid medium and incubated at 28°C for 24 h. The yeast cultures were centrifuged at 2,744 g for 10 min, and the pellet was weighed and then homogenized in 500 μl 50 mM phosphate buffer pH 7 by Bead Ruptor Elite (OMNI International, Kennesaw, GA, USA) and centrifuged. The lysate was collected and incubated with 5 μg/ml 9-HPODE at 30°C overnight. The reaction was terminated by adding an equal amount of 1 ppm 2-hydroxydecanoic acid in ethanol. LC–MS analyses of the lysates were conducted as described above.

## Results

### Nematode-Induced *LeDES* Transcript Accumulation

**Figure 1:**
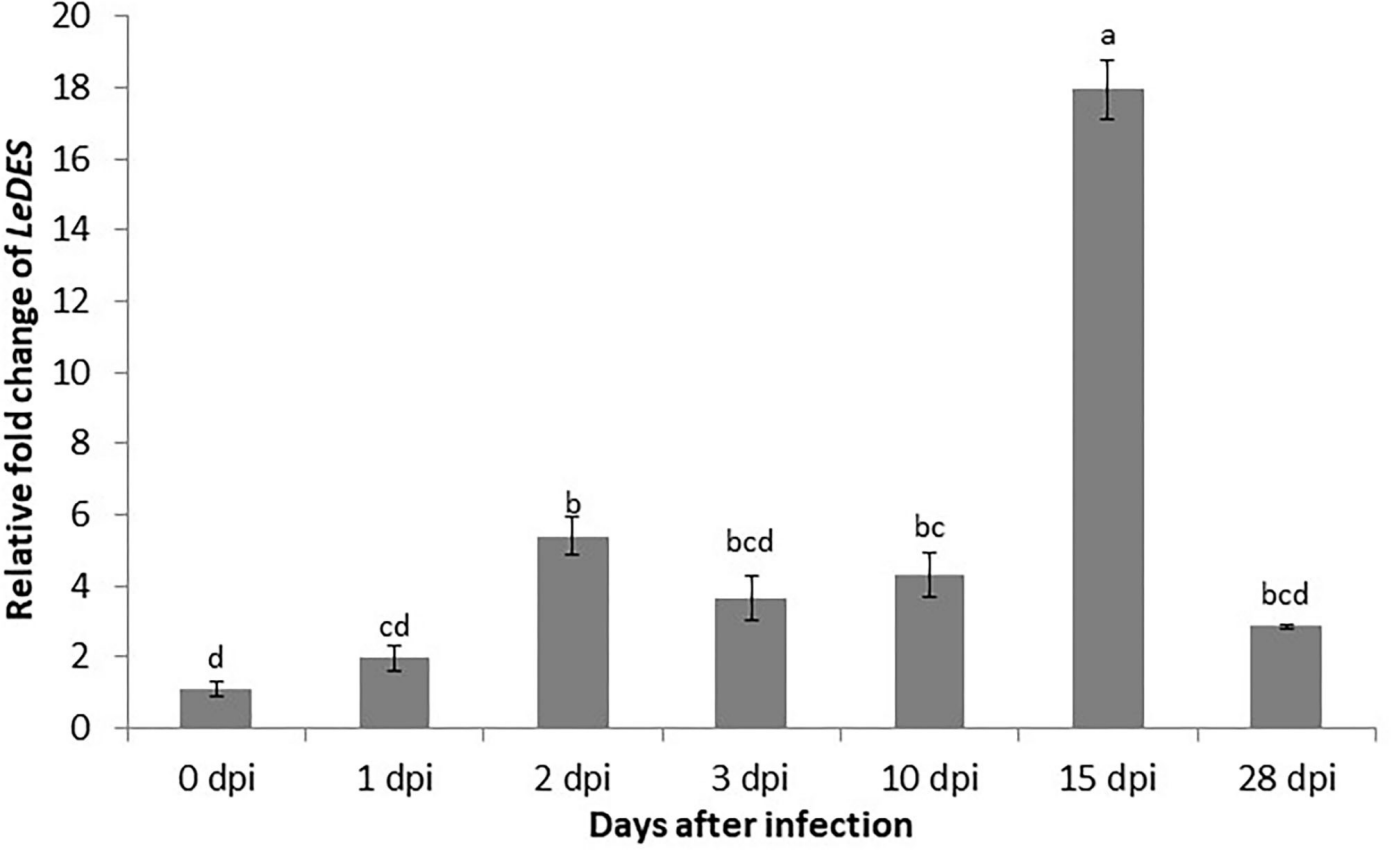
Expression pattern of *LeDES* in tomato roots during the infection time course. To investigate the transcript levels of *LeDES* upon nematode infection, total RNA was isolated from each sample at 1, 2, 3, 10, 15, and 28 dpi or without infection; RNA was subjected to qRT-PCR and normalized to β-*tubulin* as the reference gene. Two biological replicates were taken and three independent qRT-PCRs were performed per sample, resulting in a total of six replicates for statistical analyses. The graph shows the mean and SD of the amount of *LeDES* transcript relative to non-inoculated samples. Error bars correspond to SD *n* = 2 while different letters above the bars denote a significant difference (*P* ≤ 0.05, ANOVA) between different treatments as analyzed by Tukey–Kramer multiple comparison test.

To study the expression of *LeDES* in roots of tomato challenged with *M*. *javanica* J2s, qRT-PCR was performed with cDNA from root tissues collected 1, 2, 3, 10, 15, and 28 dpi. The nematodes induced activation of *LeDES*, as shown by a moderate course of increase in expression at all-time points upon inoculation (Figure 1). The relative fold changes in *LeDES* transcript in infected roots at 1, 2, 3, and 10 dpi were approximately 1.9, 5.3, 3.6, and 4.3, respectively, compared to non-inoculated roots. Transcript abundance of *LeDES* was dramatically induced at 15 dpi (17.9-fold), and was the highest for all-time points considered in this study (Figure 1). *LeDES* expression levels declined at 28 dpi, suggesting that *LeDES* has a role in the early defense mechanism induced by nematode penetration and migration, with expression peaking when the young developing galls form.

### Spatiotemporal Expression and Distribution of *LeDES* Transcripts

**Figure 2:**
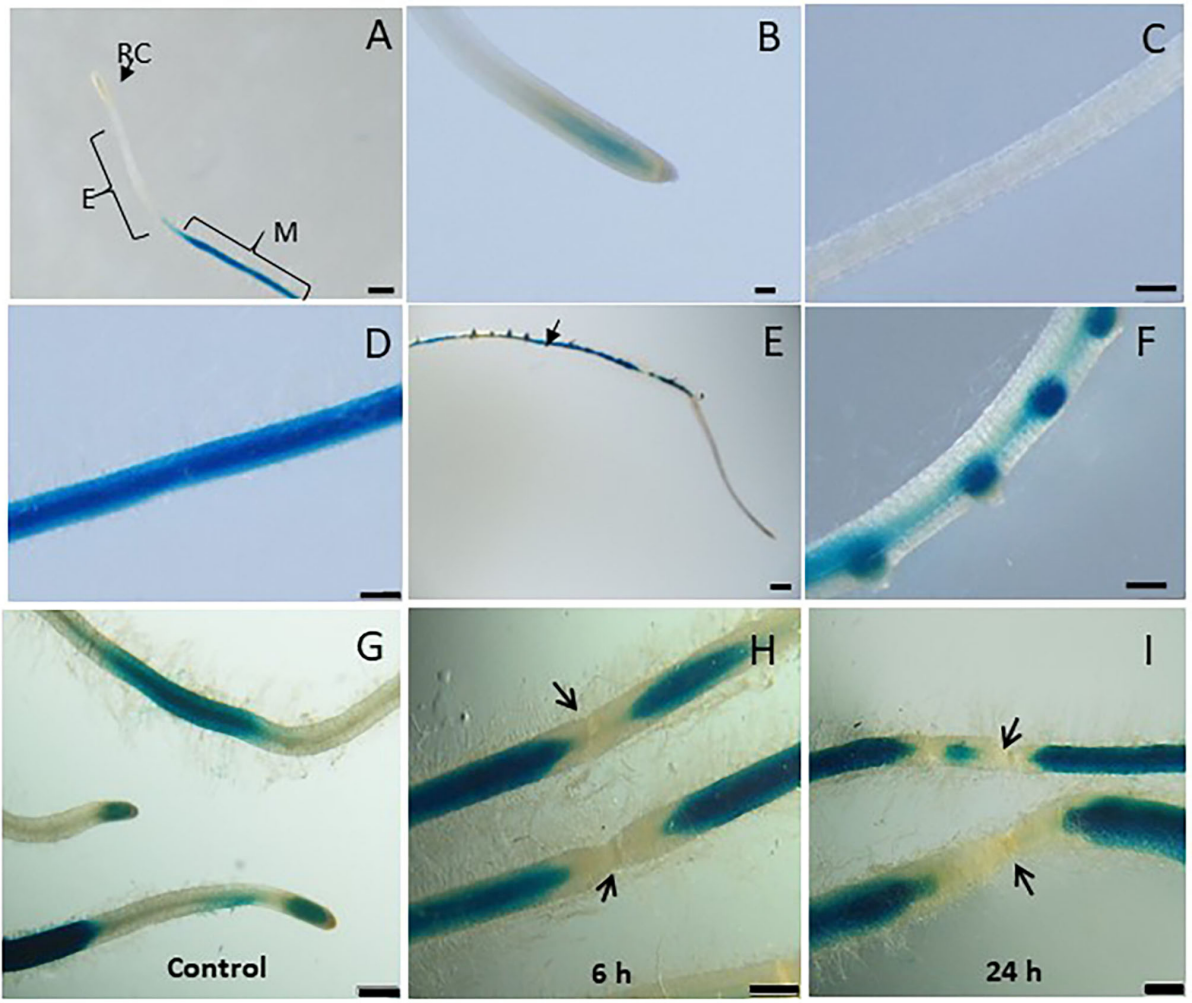
Studying basal and wound induced *LeDES* GUS expression profile. Typical expression pattern of pLeDES::GUS in non-inoculated tomato root lines. **(A)** Basal GUS activity in roots. **(B–D)** Magnified image of root cap **(B)**, elongation zone **(C)**, and maturation zone **(D)**. **(E,F)** Promoter–GUS activity observed in lateral root primordials in non-inoculated roots. RC, root cap; E, elongation zone; M, maturation zone. Bars: **(A,E)** 1 mm; **(B–D,F)** 250 μm. Expression analysis of pLeDES::GUS tomato root lines in response to wounding **(G–I)**. Histochemical GUS staining of pLeDES::GUS in control roots **(G)** and in roots 6 h **(H)** and 24 h **(I)** after wounding. Bars: **(G–I)** 1 mm. Arrows indicate site of mechanical wounding.

In order to investigate the spatiotemporal activity of LeDES promoter, the 1,602-bp sequence upstream of the ATG start site of *LeDES* was fused to the GUS reporter gene, generating a *pLeDES::GUS* construct. The *pLeDES::GUS* reporter construct was then transformed into tomato via *R. rhizogenes*-mediated root transformation. Transgenic hairy roots that emerged from the cotyledons were confirmed to be carrying the *pLeDES::GUS* construct. *pLeDES*-driven GUS expression was monitored following wounding, exogenous application of defense-related signaling molecules, and in response to nematode infection conducted at specific time points after induction, in transgenic hairy roots by histochemical staining. In the absence of any stimulus, very intense GUS staining was typically observed in the root maturation zone (Figures 2A,D), whereas no GUS activity was found in the elongation zone (Figures 2A,C); notably, a very faint GUS signal could be observed in the root apex (Figure 2B). Strong GUS activity was also associated with the lateral root primordium of the non-inoculated roots, suggesting a role for *LeDES* in genetic regulation of root growth and development (Figures 2E,F).

### Tomato *LeDES* Expression Is Repressed Upon Wounding

Mechanical damage by wounding activates defense signaling pathways and alters hormonal levels in plants, which in turn protects the plant against injury and pathogen attack (Savatin et al., 2014). *LeDES* expression upon wounding was profiled by analyzing GUS expression in *pLeDES*::GUS roots of wounded and non-wounded roots 6 and 24 h after mechanical wounding. Whereas, a typical signal was observed along the root maturation zone (Figure 2G), no LeDES promoter activity was observed localized at the wound site at either 6 h (Figure 2H) or 24 h (Figure 2I) after wounding. It is clearly observed that a complete loss of GUS staining characterize the specific site of mechanical damage as observed (arrows in Figures 2H,I) 6 and 24 h after wounding, respectively. These results suggest wound-induced local suppression of *LeDES* at the site of the mechanical damage (Figure 2).

### *LeDES* Mediates Me-SA and Auxin Phytohormone-Induced Response

**Figure 3:**
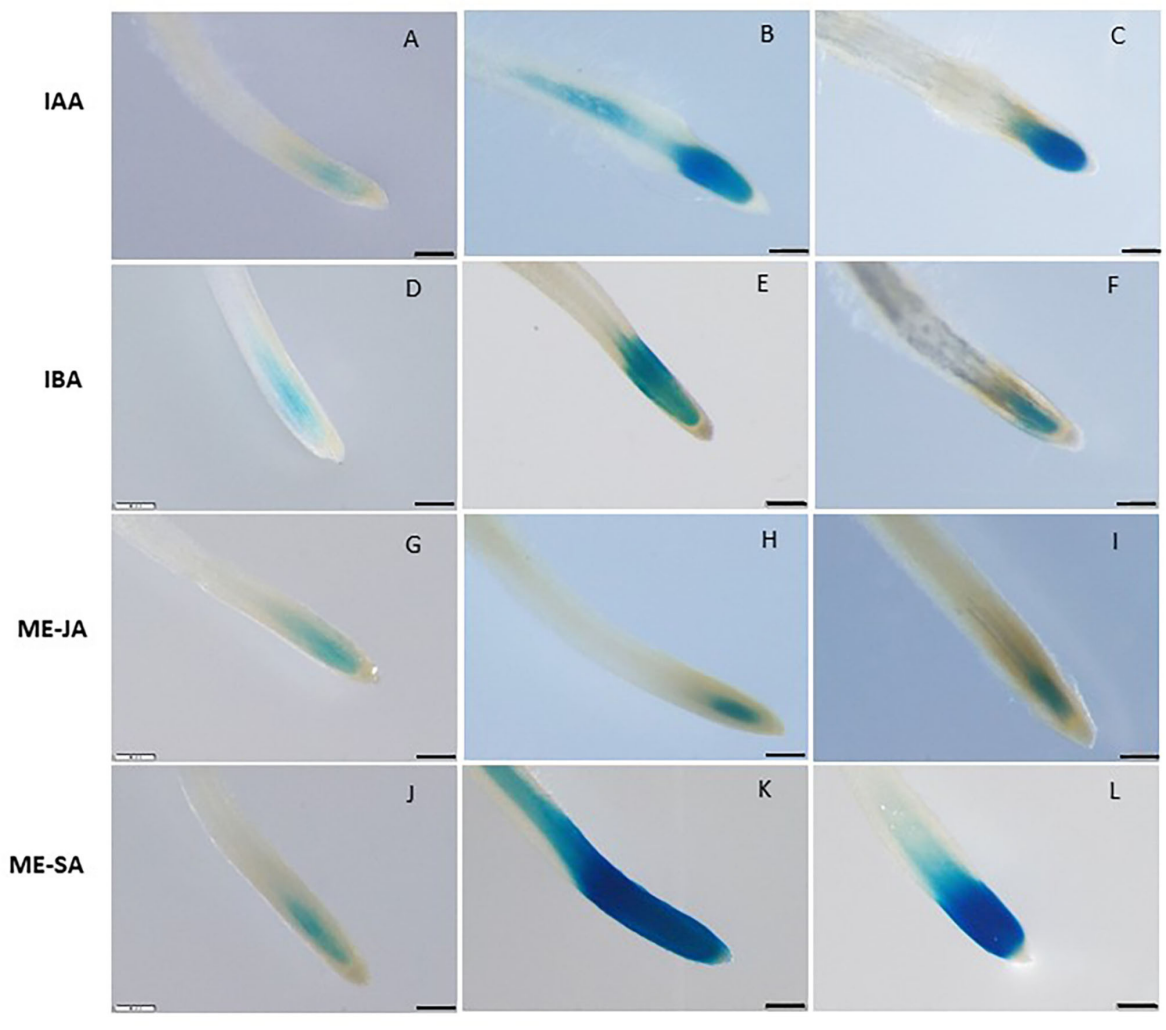
GUS staining in transgenic tomato roots carrying pLeDES::GUS following exogenous phytohormone application. One-week-old roots were subjected to GB as a control **(A,D,G,J)** or to GB containing IAA [1 and 5 μM; **(B,C)**], IBA [1 and 10 μM; **(E,F)**], Me-JA [0.01 and 0.1 mM; **(H,I)**], or Me-SA [1 and 5 mM; **(K,L)**], for 16 h. GUS staining was monitored histochemically in root tips. Figures are representative of at least three independent experiments. Bars: 250 μm.

To assess whether LeDES promoter is activated through phytohormone signaling, we used the phytohormone signaling molecules IAA, IBA, Me-JA, and Me-SA in an LeDES::GUS promoter bioassay (Figure 3). GUS staining revealed high GUS expression in the root tips 16 h after treatment with the auxins (IAA and IBA) (Figures 3B,C,E,F) compared to the negative control (Figures 3A,D), and no activation of *LeDES* promoter by Me-JA (Figures 3H,I). Notably, strong induction of the GUS reporter gene was evident after Me-SA treatment, as observed in the root tip and elongation zone (Figures 3K,L). Our results suggest that various phytohormones coregulate *LeDES* expression, highlighting its possible role in mediating defense responses triggered by biotic stress.

### Spatiotemporal Expression Pattern of *LeDES* Upon *M. javanica* Root Infection

**Figure 4:**
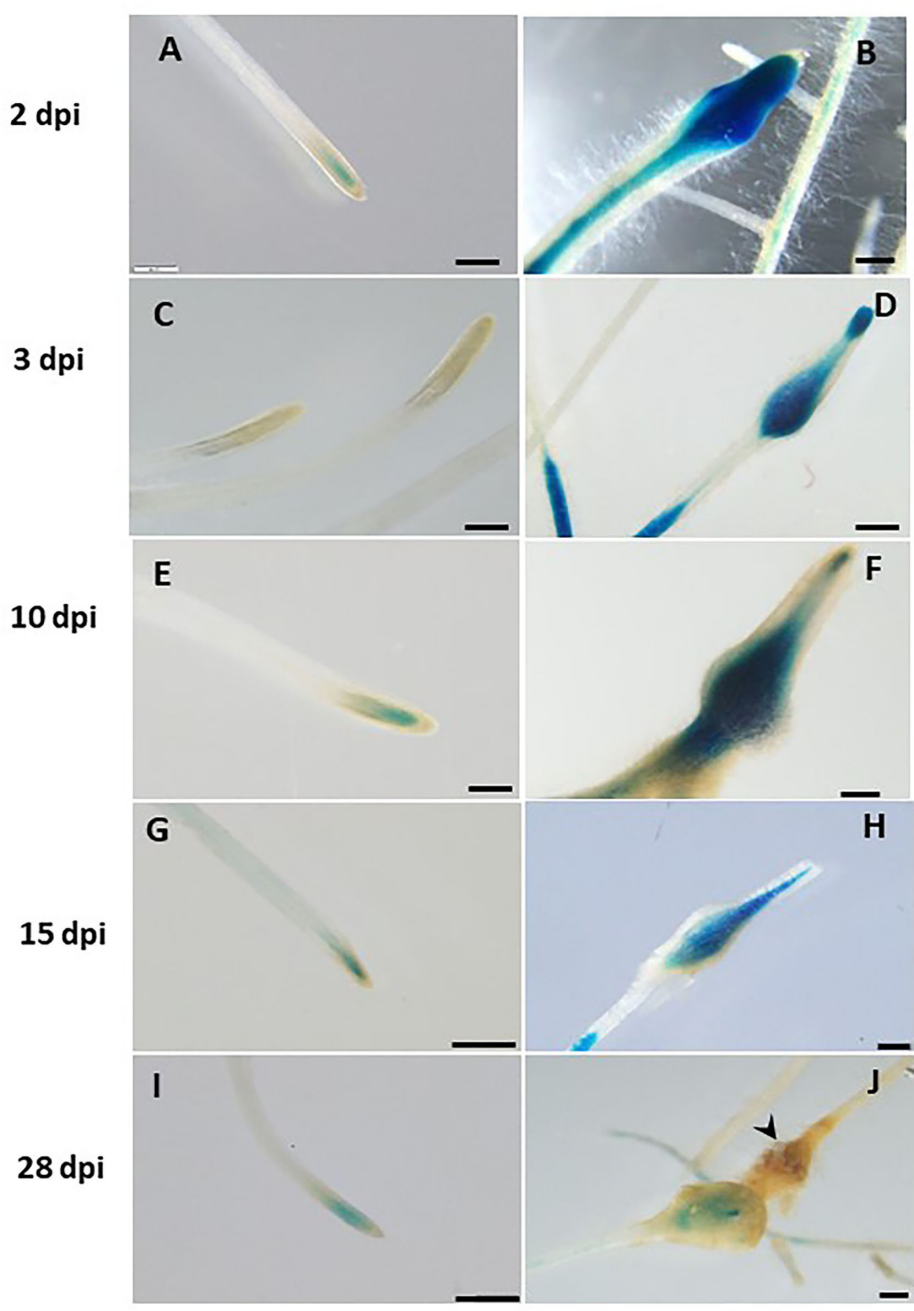
Expression-pattern analysis of pLeDES::GUS root lines during nematode infection. Non-inoculated control root demonstrated consistent GUS staining in the apical meristem (cell-division zone) **(A,C,E,G,I)**. Increased GUS staining was detected in the infected swelling site located in the elongation zone on 2 dpi **(B)**, 3 dpi **(D)**, and 10 dpi, premature developing gall **(F)**. Strong GUS staining was observed in the vasculature and vessels associated with the developed gall at 15 dpi **(H)**. At 28 dpi **(J)**, GUS staining intensity decreased and became localized specifically to the cells surrounding the developed nematode. Bars: **(A,C–E)** 250 μm; **(B,F)** 100 μm; **(G–J)** 500 μm.

Next, transcriptional activation of the GUS reporter gene driven by the LeDES promoter in the transgenic hairy roots of tomato was analyzed in a time-course analysis at 2, 3, 10, 15 and 28 dpi representing J2 (2 and 3 days), J3 and J4 (10 and 15 days) and female (28 days) stages of nematode development. Very strong staining was evident 2 and 3 dpi in the root elongation zone, the preferred site for nematode penetration, along with swelling and intensive root hair growth resulting from nematode penetration compared with non inoculated roots on the left panel (Figures 4A–D). An intense GUS signal was observed in the bulging root tissue at the initial nematode penetration site, which is part of the developing gall (Figure 4B). High promoter activity continued to be observed in the maturing galls compared with their respective controls (Figures 4E–H) at 10 (Figure 4F) and 15 (Figure 4H) dpi, mainly confined to the deformed vascular bundles (Figure 4H). Notably, a significant reduction in promoter activity was evident at 28 dpi compared with the control roots (Figures 4I,J), whereas a very mild GUS signal associated with the vascular system connected to the developed female could be observed (Figure 4J).

### Giant Cell-Specific Expression of *LeDES* Accompanying Premature Stages of *M. javanica* Infection

**Figure 5:**
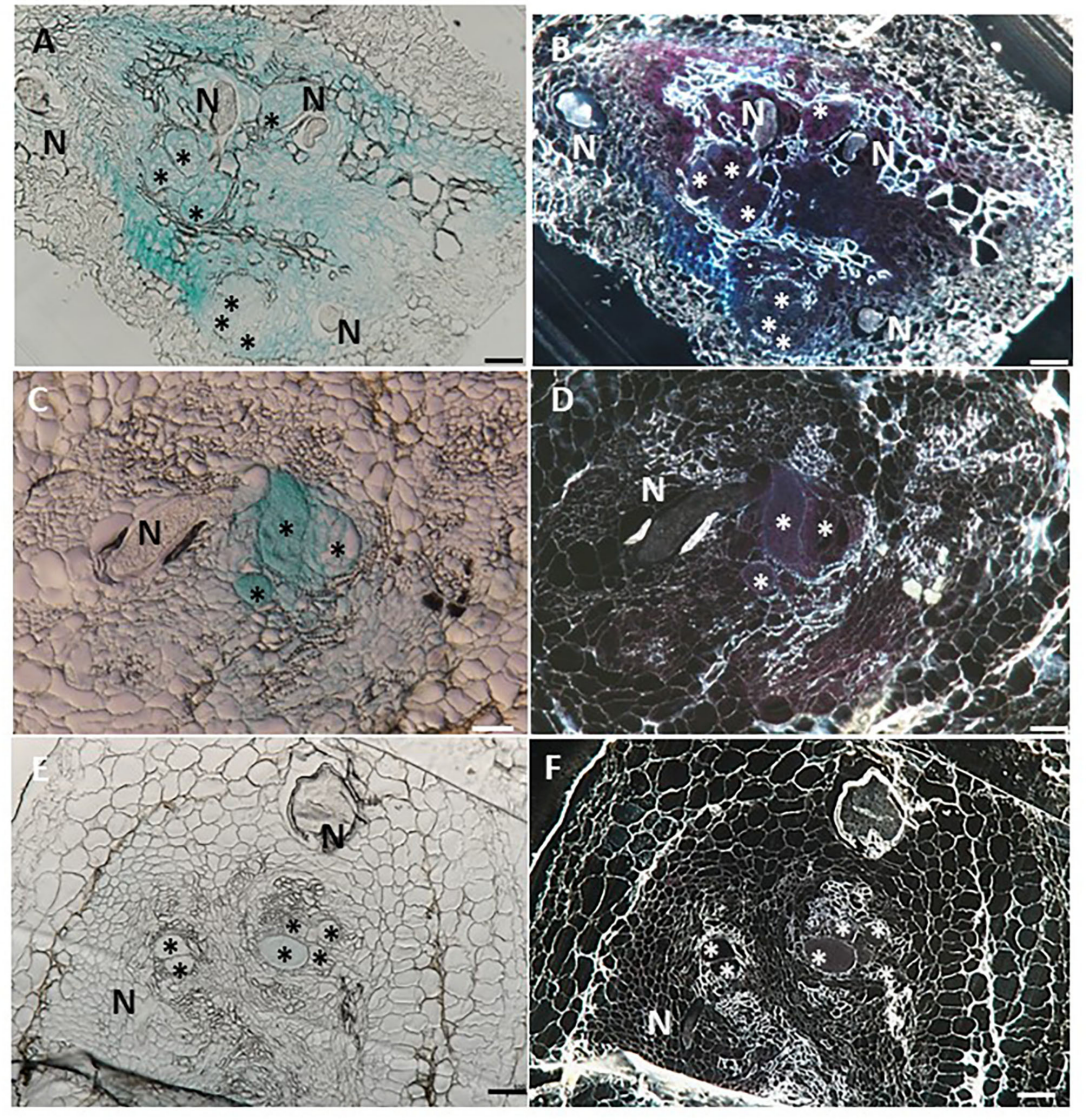
Histological GUS localization within nematode feeding site. For histological GUS localization, galls were fixed and embedded in Technovit 7100 and 3-μm-thick cross sections were analyzed using a light microscope equipped with a Nikon digital camera. All giant cells were mature and nematodes developed to the J4 stage. Histological analysis of roots expressing pLeDES::GUS on 15 dpi clearly shows GUS expression restricted to the vascular systems bordering the feeding sites, the giant cells and cells surrounding the developing female body **(A,B)**. At 28 dpi, GUS signal is observed mostly in the mature feeding site **(C,D)** and **(E,F)**. GUS staining is observed as blue color in whole mounts, and as a red precipitate in the dark field micrographs of the sections. N, female nematode body; *giant cells. Bars: 100 μm.

To investigate the spatial expression of *LeDES* at nematode feeding sites, thin sections of galls induced by *M. javanica*, 15 and 28 dpi, were prepared and analyzed. At 15 dpi, strong GUS staining showed a scattered signal distributed within the deformed stele harboring the feeding site and bounded by the endodermis cell layer, as observed under light and dark field, respectively (Figures 5A,B). At that time point, GUS signal was clearly visible in the giant cells, as well as in the surrounding xylem and phloem parenchyma cells. As infection progressed, GUS signal was significantly quenched, although it was predominantly retained within the developing giant cells (Figures 5C,D). However, as the female matured and the feeding site reached its maximum size, at 28 dpi, no GUS signal was observed in the adjacent parenchyma cells with only a very faint GUS signal in the feeding site (Figures 5E,F).

### *LeDES* Heterologous Expression in *Arabidopsis* Attenuates Nematode Development

**Figure 6:**
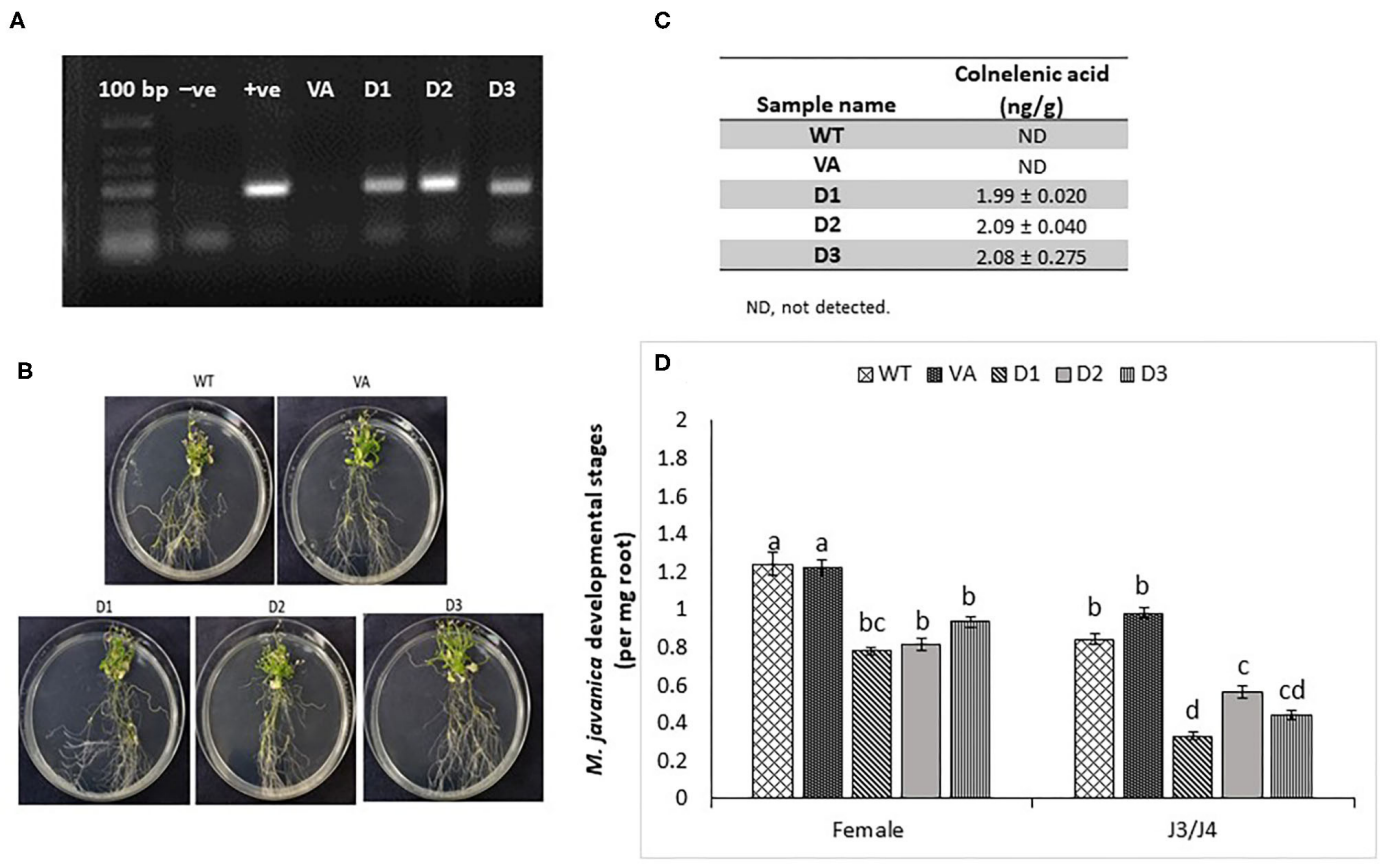
Response of *Arabidopsis* plants overexpressing tomato *LeDES* to nematode infection. **(A)** RT-PCR confirmation of *LeDES* expression with DES-specific primers. Lane 1, negative control; lane 2, cloned *LeDES* template (positive control); Lane 3, independent transgenic line transformed with empty vector; lanes 4–6, independently generated transgenic lines for LeDES **(B)** *Arabidopsis* plants expressing *LeDES* 30 dpi; root systems of all plants show normal root with no phenotypic growth changes. **(C)** Quantification of colnelenic acid produced in transgenic *Arabidopsis* root homogenates as measured by LC-MS. **(D)** Disease development in *Meloidogyne*-infected roots of transgenic *Arabidopsis* plants expressing *LeDES* compared to control lines. All plants were inoculated with 300 sterile pre-parasitic J2s and the infected roots were assessed for J3/J4 and mature female development at 28 dpi through observation under the dissecting microscope following staining with acid fuchsin dye. General Linear Mixed Model test to infection assay data was applied. Different letters above the columns indicate significant difference (*P* ≤ 0.05) among different *Arabidopsis* lines analyzed by Tukey *post-hoc* range test.

**Figure 7:**
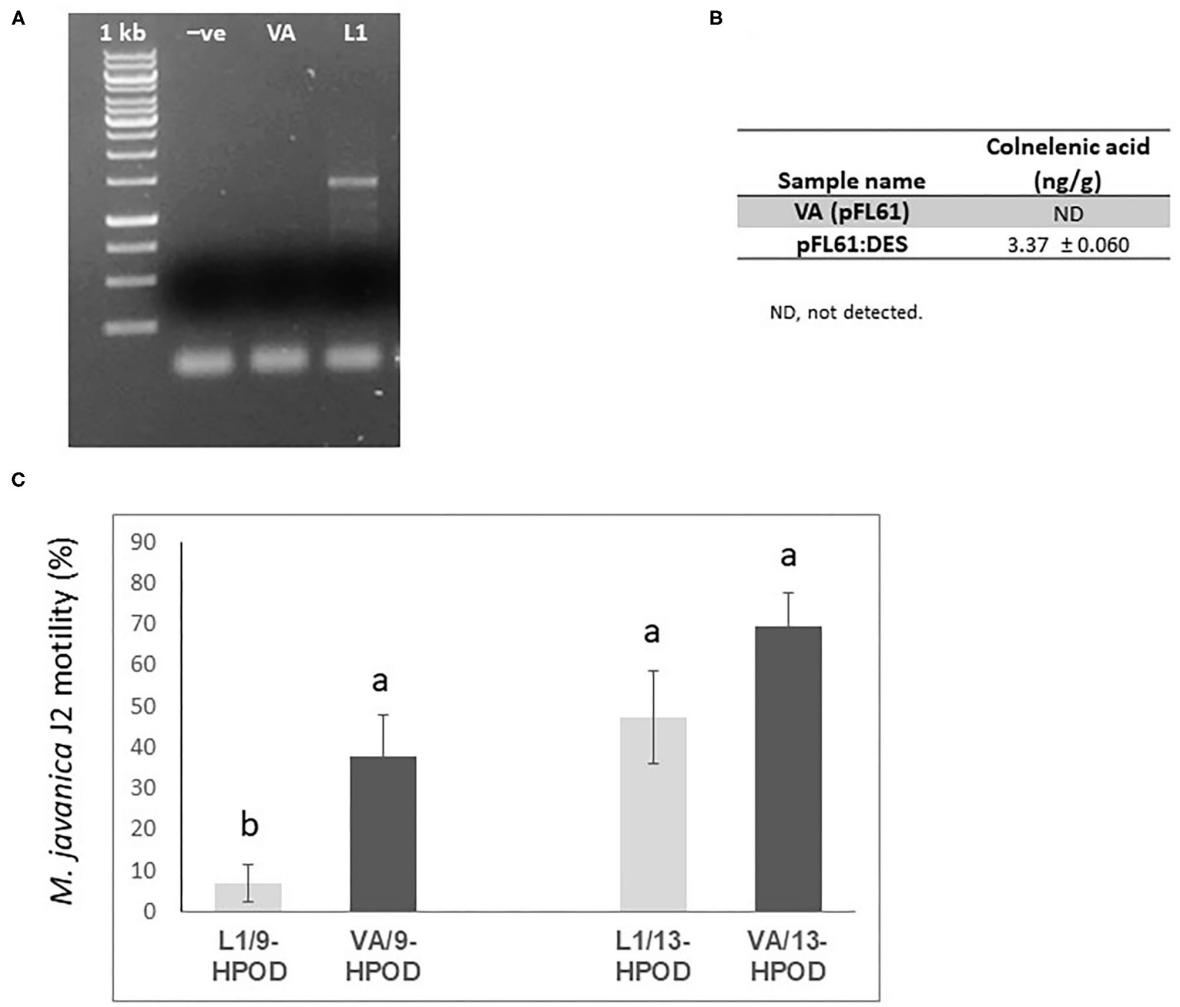
Effect of recombinant yeast expressing *LeDES* on *M. javaniva* J2 viability. **(A)** RT-PCR confirmation of *LeDES* expression in yeast. Lane 1, negative control; lane 2, transgenic yeast cells with empty vector; lane 3, transgenic yeast containing *LeDES* (L1) **(B)** Quantification of colnelenic acid production in transgenic yeast by LC–MS. Values are means of duplicate measurements. **(C)** Yeast carrying *LeDES* display bionematostatic activity. J2s were incubated in SD medium containing yeast strain and 9-HPODE or 13-HPODE for 24 h, then 300 J2s were added to the reaction for 12 h then subjected for sieving followed by microscopic observation. Different letters above the columns indicate significant difference (*P* ≤ 0.05) among the different treatments analyzed by Tukey *post-hoc* range test.

To further explore the role of *LeDES* in regulating plant responses to nematode infection, a plant binary vector containing a 1,437-bp *LeDES* ORF under the control of the CaMV 35S promoter was introduced into *A. thaliana* by floral-dip method for *Agrobacterium*-mediated transformation. From all of the independent kanamycin-resistant transgenic lines harboring the *35S:LeDES* construct, three homozygous lines (D1, D2, and D3) were validated by RT-PCR for *LeDES* heterologous expression, and used for nematode infection (Figure 6A). The transgenic plants did not show any phenotypic change in root or shoot growth compared to the WT Col-0 line (Figure 6B). To check for the presence of CnA and CA, indicative of active *DES* in the transgenic *Arabidopsis* plants, root lysates of transgenic and control plants were analyzed by LC–MS (Figure 6C). The root homogenates of transgenic and control plants were incubated with 9-HPODE and analyzed by LC–MS for the production of CnA and CA. No CnA was observed in the control plants, but it was detected in the D1, D2, and D3 transgenic lines (Figure 7C). No accumulation of CA was identified through LC–MS in either the transgenic or control WT lines. To determine the effect of *LeDES* overexpression on disease development in *Arabidopsis* transgenic lines, 10 plants each from WT, vector-only, and three transgenic lines were inoculated with 300 J2s, and the nematode developmental stages (J3/J4 and females) were monitored at 28 dpi (Figure 6D). It is commonly observed that at 28 dai, analyzed infected roots, harbor mainly J3/J4, and mature female stages with or without egg masses. While, at this late time point after infection, J2s which were suppressed or arrested at the earlier time points, will be starved to death by then, and thus barely could be visible, at 28 dai. Disease development, as indicated by number of developmental stages (J3/J4 and females) on D1, D2, and D3 and control lines (WT and vector-only) indicated that *LeDES* overexpression restricts nematode infection, and results in significantly less nematodes molting into J3/J4 stages, as observed in *LeDES* transgenic lines compared to control lines at 28 dpi. Similarly, a decrease in the number of mature females was observed in transgenic lines overexpressing *LeDES* (Figure 6D). These results indicate that heterologous expression of *LeDES* in *Arabidopsis* plants results in attenuation of nematode disease development in the roots. Infection assay data were fitted with general linear mixed model in SPSS, by keeping samples as fixed factors and repeated assays as random factor (Supplementary Table 1). Further, Tukey *post-hoc* range test was applied to check significant differences (*p* < 0.05) among different samples.

### Functional Expression of *LeDES* in *Saccharomyces cerevisiae*, Product Analysis, and Nematotoxic Acitivity

To characterize the direct activity of DVEs against *M. javanica* J2s, *LeDES* cDNA was cloned into the yeast expression vector pFL61, under the control of the constitutive PGK promoter of *S. cerevisiae* to obtain the *pFL::LeDES* construct. Then, following yeast transformation, pFL::LeDES was expressed in *S. cerevisiae* strain INVSc1. To confirm the presence and expression of *LeDES*, transgenic yeast lines were analyzed by RT-PCR, revealing accumulation of *DES* transcripts exclusively in the positive transformed line L1 (Figure 7A). Then, CnA production by the recombinant yeast was determined by incubating transgenic yeast with 9-HPODE as a substrate. LC–MS was used to detect the presence of CnA. Accumulation of 3.37 ± 0.060 ng/g CnA was observed for the L1 recombinant yeast strain, whereas no accumulation was detected in the reaction containing extract from yeast cells with pFL61 vector (Figure 7B).

Hence, recombinant yeast clones transformed with the *pFL::LeDES* construct and a negative control, the void pFL61, were used to study the direct nematotoxicfunction of DVEs against *M*. *javanica* J2s. Following 24 h incubation of recombinant yeast strains expressing either pFL::LeDES or the empty vector pFL61 with 9-HPODE or 13-HPODE *M. javanica* J2s were added to the yeast reaction for an additional 12 h, then J2 viability**/motility** was evaluated through sieving followed by microscopy observation. There was a significant difference in percent **motility** of J2s following incubation with recombinant strain pFL::LeDES and 9-HPODE (7%), compared to that with the negative control yeast carrying the empty vector pFL61 and 9-HPODE (37%) (Figure 7C). These differences in J2 **motility** were not present when 13-HPODE was used as the substrate for either of the yeast strains.

## Discussion

Oxylipins are crucial compounds in plants, playing important roles in developmental processes and in plant defense mechanisms. The involvement of oxylipins such as jasmonic acid (JA) and its derivatives in defense responses against various fungal and bacterial pathogens has been widely recognized and well-studied (Deboever et al., 2020); however, reports demonstrating the role of oxylipin-mediated signaling involved in plant–parasitic nematode interactions are scarce (Gheysen and Mitchum, 2019). We previously showed that several oxylipins exhibit significant nematicidal activity against *M. javanica*, emphasizing their crucial role in the plant's defense response against nematode parasitism (Naor et al., 2018). In another more recent study, the involvement of 9-LOX and α-dioxygenase oxylipin pathways in tomato's defense against the biotrophic RKN *M. javanica* was observed, which was also evident with the accumulation of oxylipins at earlier stages of nematode parasitism (Fitoussi et al., 2021).

Oxylipin profiling of WT roots of tobacco plants exposed to *P. parasitica* var. *nicotianae* (*Ppn* race 0) also showed preferential upregulation of the 9-LOX pathway and accumulation of the two DVEs CA and CnA (Fammartino et al., 2007). Similarly, *NtLOX1*-antisense plants were more susceptible to *P. parasitica* var. *nicotianae* (Rancé et al., 1998) along with reduced accumulation of LOX downstream pathway products, especially CA and CnA, highlighting the potential role of these oxylipins in defense responses. Other studies, analyzing the oxylipin signature in potato leaves infected with late blight disease (Weber et al., 1999) or in elicited potato cell suspension cultures (Stumpe et al., 2001) revealed the dominant induction of the DES pathway downstream of the 9-lipoxygenation of polyenoic fatty acids.

Here, we chose to study *LeDES* on the basis of our previous report which demonstrated the nematicidal activity of the DVEs CA and CnA (Naor et al., 2018). In the current study, we showed that the DVE-biosynthesis pathway is activated at certain stages of *M. javanica* infection in tomato roots, as reflected by elevated *LeDES* expression. However, differences in temporal expression were detected, where the highest expression was observed at 15 dpi. Differences in transcript accumulation in response to RKN in the tomato host suggest differential control of RKN-induced DVE biosynthesis. Colneleic acid has been shown to attenuate 9-LOX (Corey et al., 1987; Itoh and Howe, 2001; Fammartino et al., 2007), thus it can be speculated that transcription of DVE fatty acids or other oxylipin (derived from 9-LOX) biosynthesis genes may be repressed by feedback from downstream metabolites. However, the synthesis of CA and CnA is highly specific and strongly induced in certain instances, such as infection (Itoh and Howe, 2001). We therefore isolated the promoter region (1,602 bp upstream from the start codon) to obtain insight into transcriptional reprogramming of *LeDES* expression in response to RKN parasitism.

The modulation of *LeDES* expression by nematode parasitism and signal molecules related to plant defense was monitored in transgenic tomato hairy-root lines expressing the GUS reporter gene driven by the *LeDES* promoter region. Notably, in non-inoculated control roots, GUS expression was not visible in the elongation zone, but was clearly detected in the apical meristem and maturation zone. Distinct GUS staining was also observed in lateral root primordia of developing roots, emphasizing the importance of *LeDES* in regulating root development. Lateral root emergence and meristem activation are crucial developmental mechanisms in roots which can modulate root architecture in response to stress, environmental, nutritional, and endogenous factors (Signora et al., 2001; de Smet et al., 2006). Vellosillo et al. (2007) also showed that external application of CA, can, or related oxylipins to *Arabidopsis* seedlings leads to loss of apical dominance and induces the development of lateral and adventitious roots. Thus, different oxylipins produced in response to pathogen infection are probably endogenous regulators of lateral root emergence and consequently, might be involved in reprogramming root system architecture upon pathogen infection.

Nematode penetration inflicts mechanical damage to the roots, which lead to the release of Damage-Associated Molecular Patterns (DAMPs) (Choi and Klessig, 2016; Holbein et al., 2016), which in turn initiate and perpetuate the innate immune responses mediated by JA, salicylic acid (SA), ethylene, auxin, and reactive oxygen species (ROS) (Holbein et al., 2019). Many reports have shown that the defense responses activated by pathogens closely mimic those to wounding (Savatin et al., 2014). Therefore, to assess the role of *LeDES* during the early infection stage, we analyzed the effect of wounding on transgenic roots after 6 and 24 h. Expression of the GUS gene was downregulated at the wounding site, indicating that this gene is not activated as part of the DAMP-triggered immunity through cell-wall damage and may be induced by other elicitors.

Phytohormones, namely SA, ethylene, JA, abscisic acid, auxin, brassinosteroid, gibberellic acid, and cytokinin, elicit defense responses by regulating the expression of defense-related genes (Santner et al., 2009; Jaillais and Chory, 2010; Pieterse et al., 2012). Among them, JA and SA are key components in mounting defense responses in plants in response to different biotic and abiotic stresses (Denancé et al., 2013). Treatment of transgenic roots with Me-JA showed no relevant effect on GUS signal. However, application of Me-SA very strongly induced the signal in the apical meristem and elongation zone. This result fits well with the notion that SA and JA act antagonistically on each other's pathways and biosynthesis upon sensing a pathogen (Lorenzo and Solano, 2005; Pieterse et al., 2012). Plants activate mainly the SA-signaling pathway to combat biotrophic pathogens, whereas JA is involved in defense against necrotrophs (Glazebrook, 2005). Similarly, *DES* transcript in garlic leaves was induced by SA but not Me-JA application (Stumpe et al., 2008), whereas *NtDES* was not induced by SA (Fammartino et al., 2007). Considering the biotrophic nature of RKNs, we can postulate a role for SA in modulating the expression of *LeDES*. This suggests that during the process of nematode infection, the *LeDES* branch of the 9-LOX pathway participates more in the systemic defense response than in the local one through production of CA and CnA. Whether, LeDES activation is indirectly mediated through potential Nematode Associated Molecular Pattern (NAMPs) molecules, which induce SA defense-signaling pathway, that is remained to be studied.

Furthermore, treatment with IBA and IAA also induced promoter activity in the root's apical meristem, thus indicating the potential role of auxins in manipulating *LeDES* expression under basal and stress conditions. Auxins are proposed to be involved in the formation of giant cells and galls, evidenced by its increased accumulation during the early stages of feeding-site development (Karczmarek et al., 2004; Cabrera et al., 2014; Kyndt et al., 2016).

The infection tests performed with the transgenic hairy roots further confirmed the specific role of *LeDES* during the progression of nematode infection. Interestingly, during early infection stages, high GUS activity was observed in developing galls. Strong promoter activity was also evident in the middle–late infection stage (15 dpi), which then decreased (28 dpi). Hence, the *pLeDES*::GUS activation pattern was consistent with our real-time PCR results, characterized by gradual increment and then a relative reduction in expression. In sections from the 15-day galls, LeDES promoter activity was diffuse, and found throughout the vascular bundle, extending from the pericycle to the pith and including the giant cells. GUS signal became constricted to the feeding sites as the nematodes molt into the female stage, and diminished when feeding system was completely mature. This specific induction of LeDES promoter in the galls and giant cells in response to RKN infection indicates a putative role in the defense response triggered by nematode attack. Alternatively, it may be that auxin or Me-SA, which were shown to activate *LeDES*, are responsible for the observed induction.

We further cloned and expressed *LeDES* in the model plant *A. thaliana* to investigate its role in regulating plant-parasitic nematode development. Heterologous expression of the gene in *A. thaliana* conferred increased resistance against the RKN, evidenced by the attenuation in J3/J4 and female stages measured in the infected roots. Similarly, Fammartino et al. (2007) demonstrated that *NtLOX1*-antisense plants, which are compromised in their resistance to Ppn race 0, display reduced accumulation of DVEs upon inoculation. However, studies showing contradictory results have also been published. Eschen-Lippold et al. (2007) showed that infection symptoms caused by *P. infestans* were unchanged in potato lines producing a reduced amount of CnA after RNAi inhibition of pathogen-inducible DES. Furthermore, Fauconnier et al. (2008) later also showed that changes in oxylipin synthesis upon *P. infestans* infection do not correlate with resistance in potato. These differences among the differential induction of DVEs in different pathosystems might be tissue-specific, in addition to its plant- or pathogen-specific features. Thus, the induced resistance observed in the *Arabidopsis*–nematode pathosystem might be the result of differential substrate availability for DES in transgenic *Arabidopsis* plants. Nevertheless, differences between Arabidopsis and tomato pathosystems might lead to misinterpretation regards LeDES function toward RKN in tomato host, thus in future, its function should be studied by further LeDES manipulation in tomato host.

In this study, recombinant expression of *LeDES* in *S. cerevisiae* resulted in the production of measurable amounts of CnA but not CA. Exposure of *M. javanica* J2s to yeast expressing *LeDES* when 9-HPODE is the substrate, resulted in reduced J2 motility. These results, along with previous ones showing that DVE is able to attenuate motility, therefore support our findings that these are good candidate defense compounds at early time points of the infection process, further emphasizing their potential as nematostatic compounds.

## Concluding Remarks

The antimicrobial activity of different DVEs has been well-illustrated; however, there have been no reports of the involvement of DVEs in the defense response to biotic stress conditions, such as nematode infection. Our study reveals the functional role of *LeDES* by showing that this tomato gene can be overexpressed in economically important crops to improve resistance to RKNs. Furthermore, the potential of DVEs as nematostatic and signaling molecules was confirmed. However, their role in RKN resistance seems to be more complex, as they have been shown to have a nematostatic effect *in vitro*, but to play several roles *in vivo*, including as a signaling molecule mediating the plant's defense response and in root development. Their importance in regulating root architecture along with a typical nematode-responsive pattern raises new questions regarding their function while the nematode is becoming established in the roots. Further analyses, particularly of pathways that are explicitly targeted by DVEs during infection, may provide a more in-depth understanding of their specific interactions with other components of the plant's defense arsenal. These pathways' regulation should be addressed to further discriminate the relative contribution of different classes of compounds to resistance *in planta*.

## Data Availability Statement

The raw data supporting the conclusions of this article will be made available by the authors, without undue reservation.

## Author Contributions

PS and SM conceived and designed the experiment. PS conducted all plants and yeasts transformation experiments, infection studies, gene expression studies, and wrote the manuscript. AK performed GUS staining experiments, galls thin sectioning, and microscopy work. NF constructed the LeDES promter:GUS plasmid. MC-W and PS performed the oxylipins analysis. PB performed the Nematicidal activity bioassay. MB is a key person in this manuscript as all the plants required for the experiments were as a result of his constant greenhouse care. All authors made substantial contributions to the final text and read and approved the final manuscript.

## Conflict of Interest

The authors declare that the research was conducted in the absence of any commercial or financial relationships that could be construed as a potential conflict of interest.

## Publisher's Note

All claims expressed in this article are solely those of the authors and do not necessarily represent those of their affiliated organizations, or those of the publisher, the editors and the reviewers. Any product that may be evaluated in this article, or claim that may be made by its manufacturer, is not guaranteed or endorsed by the publisher.
