# Supplementary material for: Tomato Divinyl Ether-Biosynthesis Pathway Is Implicated in Modulating of Root-Knot Nematode Meloidogyne javanica's Parasitic Ability
Source: Front Plant Sci. 2021 Aug 25;12:670772. doi: 10.3389/fpls.2021.670772 (PMC8424051; doi:10.3389/fpls.2021.670772)
Supplement: Supplementary file 1 [file Table_1.DOCX]

**Supplementary Table 1.** General Linear Mixed Model Test Results of Arabidopsis Infection assay

| **Tests of Between-Subjects Effects** | | | | | | | |
| --- | --- | --- | --- | --- | --- | --- | --- |
| Dependent Variable: J3/J4 | | | | | | | |
| Source | | Type III Sum of Squares | df | Mean Square | F | P value* | η^2^ |
| J3/J4(Intercept) | Hypothesis | 37.210 | 1 | 37.210 | 633.260 | 0.002 | 0.997 |
|  | Error | 0.118 | 2 | 0.059 |  |  |  |
| Samples (WT/VA/D1-D3) | Hypothesis | 5.483 | 4 | 1.371 | 69.902 | 0.000 | 0.972 |
|  | Error | 0.157 | 8 | 0.020 |  |  |  |
| Assays | Hypothesis | 0.118 | 2 | 0.059 | 3.000 | 0.106 | 0.428 |
|  | Error | 0.157 | 8.027 | 0.020 |  |  |  |
| Samples × Assays | Hypothesis | 0.157 | 8 | 0.020 | 1.646 | 0.125 | 0.144 |
|  | Error | 0.929 | 78 | 0.012 |  |  |  |

*P value < 0.05 considered significant

η^2^=effect size

| **Tests of Between-Subjects Effects** | | | | | | | |
| --- | --- | --- | --- | --- | --- | --- | --- |
| Dependent Variable: Female | | | | | | | |
| Source | | Type III Sum of Squares | df | Mean Square | F | P value* | η^2^ |
| Female (Intercept) | Hypothesis | 92.734 | 1 | 92.734 | 3033.084 | 0.000 | 0.999 |
|  | Error | 0.061 | 2 | 0.031 |  |  |  |
| Samples (WT/VA/D1-D3) | Hypothesis | 3.504 | 4 | 0.876 | 115.772 | 0.000 | 0.983 |
|  | Error | 0.061 | 8 | 0.008 |  |  |  |
| Assays | Hypothesis | 0.061 | 2 | 0.031 | 4.014 | 0.061 | 0.496 |
|  | Error | 0.062 | 8.150 | 0.008 |  |  |  |
| Samples × Assays | Hypothesis | 0.061 | 8 | 0.008 | 0.297 | 0.965 | 0.030 |
|  | Error | 1.990 | 78 | 0.026 |  |  |  |

*P value < 0.05 considered significant

η^2^=effect size
